# RNA-Seq Analysis Reveals Different Dynamics of Differentiation of Human Dermis- and Adipose-Derived Stromal Stem Cells

**DOI:** 10.1371/journal.pone.0038833

**Published:** 2012-06-19

**Authors:** Kersti Jääger, Saiful Islam, Pawel Zajac, Sten Linnarsson, Toomas Neuman

**Affiliations:** 1 Institute of Gene Technology, Tallinn University of Technology, Tallinn, Estonia; 2 Cellin Technologies LLC, Tallinn, Estonia; 3 Department of Medical Biochemistry and Biophysics, Karolinska Institutet, Stockholm, Sweden; 4 Protobios LLC, Tallinn, Estonia; RWTH Aachen University Medical School, Germany

## Abstract

**Background:**

Tissue regeneration and recovery in the adult body depends on self-renewal and differentiation of stem and progenitor cells. Mesenchymal stem cells (MSCs) that have the ability to differentiate into various cell types, have been isolated from the stromal fraction of virtually all tissues. However, little is known about the true identity of MSCs. MSC populations exhibit great tissue-, location- and patient-specific variation in gene expression and are heterogeneous in cell composition.

**Methodology/Principal Findings:**

Our aim was to analyze the dynamics of differentiation of two closely related stromal cell types, adipose tissue-derived MSCs (AdMSCs) and dermal fibroblasts (FBs) along adipogenic, osteogenic and chondrogenic lineages using multiplex RNA-seq technology. We found that undifferentiated donor-matched AdMSCs and FBs are distinct populations that stay different upon differentiation into adipocytes, osteoblasts and chondrocytes. The changes in lineage-specific gene expression occur early in differentiation and persist over time in both AdMSCs and FBs. Further, AdMSCs and FBs exhibit similar dynamics of adipogenic and osteogenic differentiation but different dynamics of chondrogenic differentiation.

**Conclusions/Significance:**

Our findings suggest that stromal stem cells including AdMSCs and dermal FBs exploit different molecular mechanisms of differentiation to reach a common cell fate. The early mechanisms of differentiation are lineage-specific and are similar for adipogenic and osteogenic differentiation but are distinct for chondrogenic differentiation between AdMSCs and FBs.

## Introduction

Tissue regeneration is dependent on progenitor cells that self-renew and differentiate into different cell types with specialized functions. Mesenchymal stem cells (MSCs) have been isolated from many different adult organs and tissues including skin, lung, liver and fat [1]–[4]. *In vitro* studies have demonstrated that MSCs can be expanded in culture and differentiated into several cell types under appropriate conditions. In addition to fat, bone and cartilage cells, MSCs have been demonstrated to give rise to muscle and nerve cells *in vitro* [4]–[7].

In contrast, differentiation of dermal fibroblasts (FBs) into various mesodermal cell types under similar conditions has produced contradictory results. In some experimental settings FBs were shown to lack multilineage differentiation potential [8], [9], whereas other reports show that FBs and MSCs can be equally differentiated into several types of mesodermal cells [10]–[13]. Also, we have previously shown that dermal FBs and adipose tissue-derived MSCs (AdMSCs) originating from the same donors both differentiate into osteoblasts and adipocytes [14]. The immunophenotypes of MSCs and FBs are similar based on numerous surface markers currently used to identify MSCs. Both cell types express cell surface antigens CD73, CD90 and CD105 [9], [10], [13].

The molecular characterization of MSCs is hampered by the lack of biomarkers that would allow their selective isolation from different tissue sources with heterogeneity of cell populations. MSCs are currently isolated as plastic-adherent cells with fibroblast-like morphology that can be differentiated into several mesodermal cell types [15]. These parameters are not sufficient to discriminate MSCs from FBs and do not aid in the understanding of the identity of these cell types. Another problem is comparison of different types of stromal cells including dermal FBs and AdMSCs isolated from individuals with different genetic backgrounds. This could lead to differences in gene expression patterns and cellular functions that cannot directly be associated with distinct cell identities.

**Figure 1:**
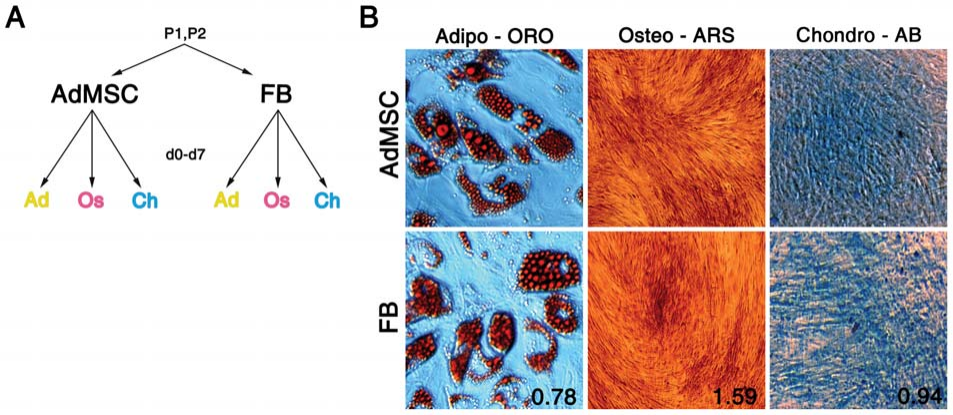
Cell differentiation. A) AdMSCs and FBs were isolated from two patients (P1, P2) and differentiated towards adipocytes, osteoblasts and chondrocytes. RNA was isolated on days 0–7 during differentiation and the resulting 96 RNA samples were used to generate single sequencing library for gene expression analysis. B) *In vitro* differentiation of AdMSCs (upper panel) and FBs (lower panel) was confirmed by ORO staining of adipocyte, ARS staining of osteoblast and AB staining of chondrocyte cultures on day 14 upon induction of differentiation. The quantified stainings of FBs are represented relative to AdMSCs (lower panel; AdMSC = 1). Abbreviations: P, patient; AdMSC, adipose-derived mesenchymal stem cell; FB, fibroblast; Ad, adipocyte; Os, osteoblast; Ch, chondrocyte; d, day; ORO, Oil Red O; ARS, Alizarin Red S; AB, Alcian Blue.

Here we aimed to analyze the transcriptome profiles of several differentiated cells starting from AdMSCs and dermal FBs obtained from two matching donors and differentiated under similar experimental conditions towards adipocytes, osteoblasts and chondrocytes (Figure 1A). RNA-seq-derived gene expression data was compared by a multi-group ANOVA, and differences between groups other than those used in the ANOVA were then visualized using principal component analysis (PCA). To our knowledge, this is the first study to compare the dynamics of differentiation of AdMSCs and FBs into three mesodermal cell types on global scale.

## Results

### Transcriptome Profiles of Multipotent AdMSCs and FBs

#### Both AdMSCs and FBs exhibit adipo-, osteo- and chondrogenic developmental potential

Prior to the analysis of the global gene expression profiles of differentiating AdMSCs and FBs in more detail, we aimed to verify that both of these cell populations exhibit multipotency. Cells derived from two donors were plated at 72 h prior to addition of differentiation media and cultivated for 14 days until analysis (see Materials and Methods). *In vitro* differentiation of AdMSCs and FBs was confirmed by detection of formation of lipid droplets with Oil Red O staining (ORO, adipocytes), matrix mineralization with Alizarin Red S staining (ARS, osteoblasts) or formation of proteoglycan-rich matrix with Alcian Blue staining (AB, chondrocytes). Induced AdMSCs and FBs (from both donors) differentiated into cells with positive staining for ORO, ARS and AB confirming the similar developmental capacity of these cell types (Figure 1B). Quantification of lineage-specific staining showed that the differentiation potential of FBs and AdMSCs is indeed comparable (Figure 1B, lower panel shows staining intensities of FBs relative to AdMSCs). This analysis together with previous reports [10], [13], [14] confirms that multipotency is not solely restricted to AdMSCs but is also characteristic to fibroblasts. Immunophenotyping showed that AdMSCs and FBs from both donors expressed cell surface antigens CD73 and CD105 (data not shown).

#### Global transcriptome profiling reveals AdMSC- and FB- specific gene expression patterns

For transcriptome analysis, cells were treated as described in Materials and Methods section and RNA was isolated every 24 h on days 0–7 upon adipogenic, osteogenic and chondrogenic differentiation. Single sequencing library was then generated from the resulting 96 RNA samples (Table S1) using a method by Islam *et al*, 2011 [16] with slight modifications (see Materials and Methods). Deep sequencing yielded 45 million mapped reads and 60% of those mapped to known transcripts in the human genome. 9000 most highly expressed features with normalized hit values ranging from 6.25 to 23 437.5 transcripts per million (t.p.m), that cover 99% of the transcripts and include both the most highly expressed genes as well as rare transcripts, were used in gene expression analysis. Five samples were removed from the analysis (Table S1) due to unsatisfactory RNA quality (total read number was below 0.01% of all samples). Each time point in the assay was represented by a single replicate except for day 0 that was sequenced in triplicate (three different RNAs). Each sample from the total of 91 was annotated according to its tissue of origin (AdMSC or FB), patient of origin, cell type and time point.

**Figure 2:**
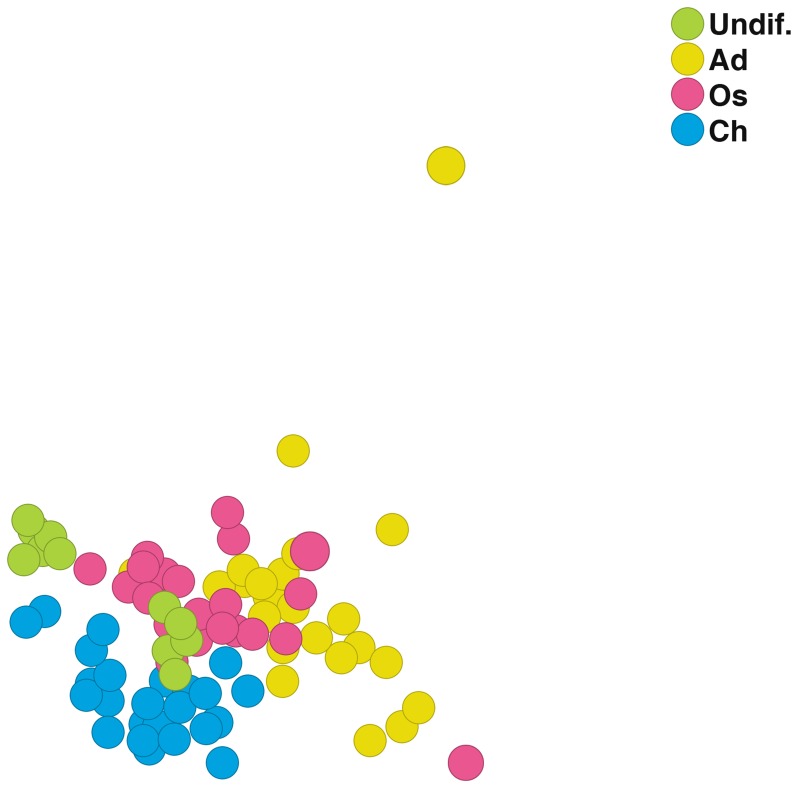
Principal component analysis (PCA) of non-filtered data. 9000 most highly expressed genes were visualized by PCA based on cell type (undifferentiated, adipocytes, osteoblasts and chondrocytes) without prior statistical filtering. Different cell types cluster together upon PCA. Abbreviations: Undif., undifferentiated; Ad, adipocyte; Os, osteoblast; Ch, chondrocyte.

First, we analyzed how different samples are connected to each other using principal component analysis (PCA) on complete gene expression data without prior statistical filtering (Figure 2). The circles in Figure 2 represent individual samples and are visualized according to cell type (undifferentiated cells, adipocytes, osteoblasts and chondrocytes). PCA shows that samples belonging to the same cell group cluster together, except for a few adipocyte-samples and one osteoblast-sample that stay apart from the clusters. Interestingly, undifferentiated cells make up two distinct clusters. The analysis shows that the RNA-seq-derived transriptome profiles are characteristic to different cell types.

The analysis above was performed with unfiltered data. However, PCA can be used to visualize filtered data. We used multi-group ANOVA to compare gene expression between defined groups and then used the ANOVA-filtered data in subsequent PCAs to visualize differences between other groups (not between those used in the ANOVA). The genes were selected for ANOVA based on false discovery rate (FDR) to control the effects for multiple testing. The step-wise filtering and vizualisation of the data was performed with Qlucore Omics Explorer.

**Figure 3:**
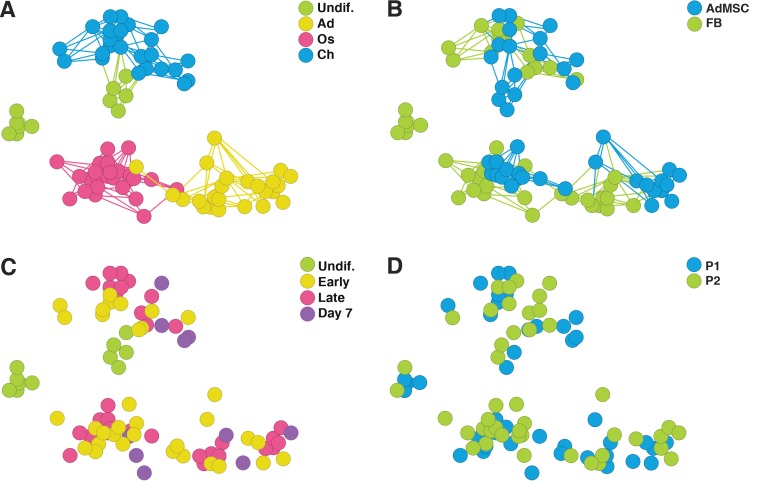
PCA of cell type-specific gene expression. 9000 most highly expressed genes were analyzed by multi-group ANOVA to find differentially expressed genes between cell types: undifferentiated AdMSCs and FBs, and AdMSC- and FB-derived adipocytes, osteoblasts and chondrocytes using false discovery rate (FDR) of 0.1%. PCA of the resulting 792 genes was used to visualize the relationship of the samples based on annotations such as A) cell type, B) cell origin (AdMSC or FB), C) time groups of differentiation and D) patient. Each circle represents one sample, and is connected by edges to four other most closely related samples in A and B. The same genes that separate different cell types, also separate undifferentiated AdMSCs and FBs and are regulated over time with no differences between patients. However, AdMSCs and FBs retain characteristic gene expression even in the differentiated state. Abbreviations: Undif., undifferentiated; Ad, adipocyte; Os, osteoblast; Ch, chondrocyte; AdMSC, adipose-derived mesenchymal stem cell; FB, fibroblast; P, patient.

Next, we analyzed how different cell types (undifferentiated cells, adipocytes, osteoblasts and chondrocytes) are related to each other based on filtered differences in gene expression. A multi-group ANOVA with a FDR of 0.1% recovered 792 differentially expressed genes between different cell types. PCA was then used to visualize the relationship of the individual samples (Figure 3). The edges in Figure 3 connect each sample with the four other most closely related samples (in A and B). The same PCA plot was used to visualize the samples based on different annotations such as cell type, AdMSC or FB, time group (‘early’ including days 1–3 and ‘late’ including days 4–6) and patient of origin. Cell type-based visualization (Figure 3A) shows that 792 genes clearly generate clusters from samples belonging to the same cell group. This is unsurprising, since the ANOVA selected for genes that distinguish between cell types. However, the samples also show a clear separation by time group, demonstrating that those genes that distinguish cell types were also differentially regulated over time. Importantly, undifferentiated cells make up two distinct clusters. One of them (FB) locates separately with no connections to other clusters, whereas the other (AdMSC) is closely connected to chondrocytes (Figure 3B). Upon differentiation, clusters of AdMSCs and FBs become close already on day 1 and stay close in all time groups (Figure 3C). Interestingly, despite the loss of initial differences between AdMSCs and FBs upon differentiation, AdMSC- and FB- specific sub-clusters still remain apparent within adipocytes, osteoblasts and chondrocytes. The samples originating from two patients were intermingled, verifying the reproducible and patient-independent formation of cell type-specific clusters (Figure 3D). ANOVA between the two patients over the total expression data (9000 genes) identified no genes that were significantly (FDR of 1%) differently expressed between the individuals. Hence, the differences between cell types overwhelm any differences between these donors. Since different media was used to cultivate undifferentiated AdMSCs and FBs at optimal conditions (media was chosen so that AdMSCs and FBs exhibited similar growth rate), it cannot be excluded that some of the differences in gene expression between AdMSCs and FBs arise from the different media compositions. Taken together, these data show that AdMSCs and FBs represent initially distinct populations with regard to the expression of developmentally regulated genes, and they also stay subtly distinct in the differentiated state. The *in vitro* development of mature cell types usually takes 2–4 weeks. It is thus possible that the differences between AdMSCs and FBs that are evident after one week of differentiation may disappear after longer differentiation.

### Undifferentiated AdMSCs and FBs are Different

#### AdMSCs and FBs exhibit different gene expression patterns in the undifferentiated state

**Figure 4:**
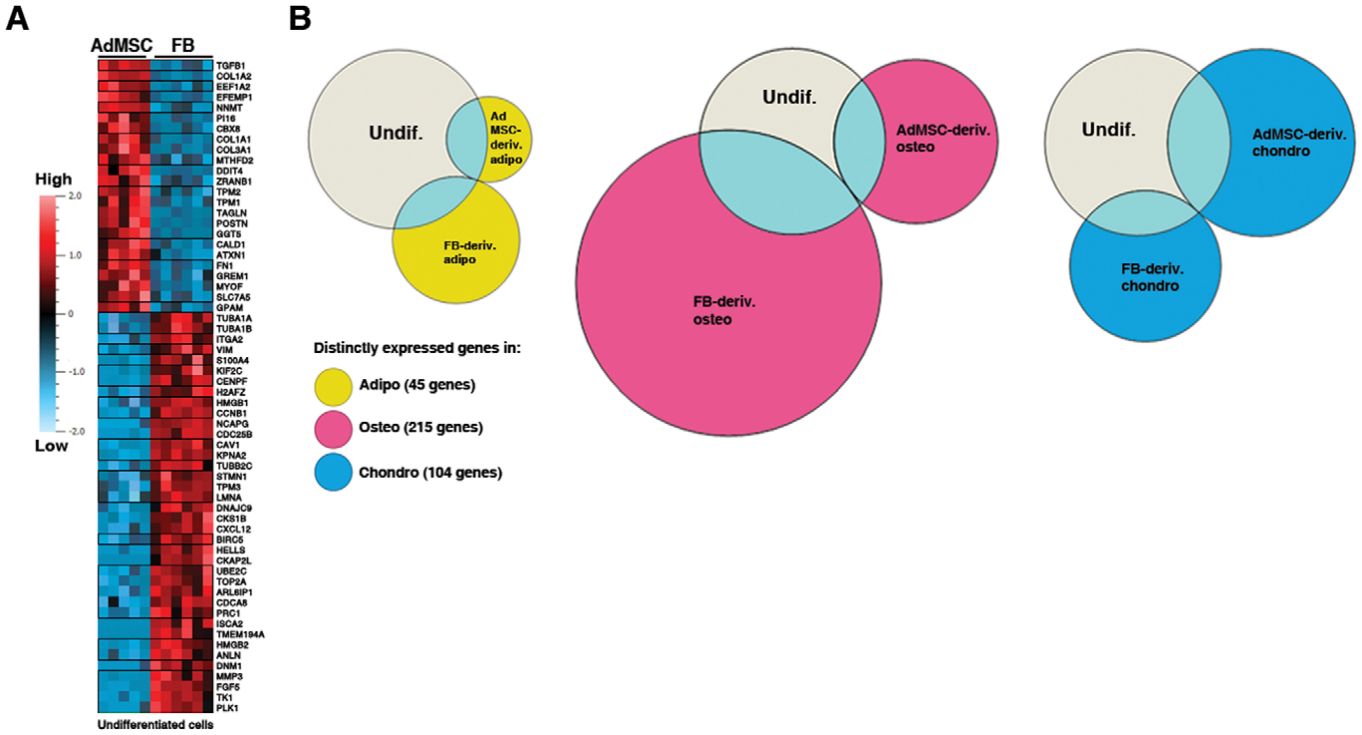
Differences in gene expression of AdMSCs and FBs. A) ANOVA with FDR of 1% between undifferentiated AdMSCs (5 replicates) and FBs (6 replicates) recovered 62 differentially expressed genes, 24 with higher and 38 with lower expression in AdMSCs than FBs. The scale shows the up (light red) or down regulation (light blue) in standard deviations from the mean expression for each gene. B) Comparison of differentially expressed genes between AdMSCs and FBs in the undifferentiated state (light grey) and in AdMSC- and FB-derived adipocytes, osteoblasts and chondrocytes using Venn diagram. Many genes remain (light blue) and many differentiation-related genes become (yellow, pink or blue) differentially expressed in AdMSC- and FB-derived differentiated cells. Abbreviations: AdMSC, adipose-derived mesenchymal stem cell; FB, fibroblast; Undif., undifferentiated; Adipo, adipocyte; Osteo, osteoblast; Chondro, chondrocyte; deriv., derived.

The observation that undifferentiated AdMSCs and FBs clustered separately based on the expression of 792 lineage-specific genes raised the question how different are AdMSCs and FBs before differentiation. Heat map-view of differentially expressed genes (including 9000 genes) was generated using all replicate samples (5 of AdMSCs and 6 of FBs). The scale in Figure 4A shows the up (red) or down regulation (blue) in standard deviations from the mean expression for each gene. Altogether 62 genes were found to have significantly (FDR of 1%) different expression between AdMSCs and FBs, 38 with higher and 24 with lower expression in FBs than in AdMSCs. ANOVA with five times higher false discovery rate (5%) resulted in 116 more genes (Figure S1). The relatively small number of differentially expressed genes between AdMSCs and FBs could be explained by their common mesodermal origin that probably determines the general transcription profile of the cells. Also, in cell culture, AdMSCs grow as fibroblast-like cells and exhibit morphology similar to FBs, so that the substantial overlap in gene expression patterns between the cells can be expected.

### Genes with Various Functions are Distinctly Expressed between AdMSCs and FBs

We then asked whether 62 differentially expressed genes represent functional differences between AdMSCs and FBs. These genes were grouped according to their known function that resulted in six predominant classes (Table 1). 20 genes out of 38 with higher expression in undifferentiated FBs than AdMSCs are related to cell cycle regulation, more specifically to G2/M phase of the cell cycle. Also, the majority of genes involved in the regulation of cytoskeleton stability and in cellular signaling pathways (cell motility - *S100A*4, vesicular trafficking - *CAV1, DNM1*) had higher expression in FBs compared to AdMSCs. However, expression of genes associated with either BMP (*GREM1*), VEGF (*MYOF*) or Wnt signaling (*ZRANB1*) was significantly higher in AdMSCs compared to FBs. Most of the genes that participate in the biosynthetic processes or in the regulation of extracellular matrix organization and adhesion had higher expression in AdMSCs than FBs. Interestingly, we identified high expression of developmentally important gene chromobox homolog 8 (*CBX8*) in AdMSCs but not in FBs (Table 1). CBX8 is an essential component of the Polycomb group (PcG) multiprotein PRC1 complex that is required to maintain transcriptionally repressive state of many genes, including Hox genes, throughout development [17]. Whether CBX8 has any functional role in determining the differences between AdMSCs and FBs remains to be elucidated in future studies. Together, our results suggest that despite the similar general characteristics of AdMSCs and FBs, the gene expression profiles are distinct due to differences in expression of genes involved in the regulation of cell cycle and developmental processes and also in the structural organization of the cell.

**Table 1:** Distinctly expressed genes between undifferentiated AdMSCs and FBs (based on ANOVA with FDR of 1%).

	Genes with higher expression in:	
	FBs	AdMSCs
**Cell cycle**	ANLN, BIRC5, CCNB1, CDC25B, CDCA8, CENPF, CKS1B,H2AFZ, HELLS, HMGB1, HMGB2, KIF2C, KPNA2, LMNA, NCAPG,PLK1, PRC1, TK1, TOP2A, UBE2C	
**Cytoskeleton**	CKAP2L, STMN1, TPM3, TUBA1A, TUBA1B, TUBB2C, VIM	CALD1, TAGLN, TPM1, TPM2
**Extracellular matrix** **and adhesion**	ITGA2, MMP3	COL1A1, COL1A2, COL1A3, EFEMP1, FN1, POSTN, TGFBI
**Biosynthesis**	ISCA2	EEF1A1, GGT5, GPAM, MTHFD2, NNMT, PI16, SLC7A5
**Signal transduction**	CAV1, CXCL12, DNM1, FGF5, S100A4	GREM1, MYOF, ZRANB1
**Development**		CBX8

#### AdMSCs are more similar to chondrocytes than FBs

The observation that AdMSCs are closely connected to chondrocytes (Figure 3A and B, PCA of developmentally regulated genes) reveals important aspects of differences between AdMSCs and FBs. In search for similarities between AdMSCs and chondrocytes, 792 differentially expressed genes were analyzed to identify genes that are highly expressed in AdMSCs and chondrocytes but not in FBs. We compared the expression of genes in undifferentiated cells and in day 1 AdMSC- and FB-derived chondrocytes, since gene expression patterns become similar at later time points of chondrogenic differentiation. As few as 23 genes were found to have higher expression in AdMSCs and AdMSC- and FB-derived chondrocytes compared with undifferentiated FBs (Table 2). The genes were grouped into five functional classes including cytoskeleton, extracellular matrix and adhesion, processes of biosynthesis, signal transduction and development. The majority of genes that were enriched in AdMSCs and chondrocytes encode ribosomal proteins and function in protein biosynthesis. Also, structural components of the cytoskeleton and genes that regulate ECM-mediated cell signaling and adhesion showed higher expression in AdMSCs and chondrocytes compared to FBs. Two genes, *DACT1* (Wnt signaling) and *PDLIM7* (BMP6 signaling) involved in developmental processes were common to AdMSCs and chondrocytes. Both of these pathways play important role in cartilage development [18], [19]. Our data show that different cell types have different expression of lineage-specific genes (Figure 3A) and suggests that unlike FBs, undifferentiated AdMSCs may share functional similarities with chondrocytes.

**Table 2:** The list of genes that are highly expressed in AdMSCs and chondrocytes but not in FBs.

Cytoskeleton	FRMD6, TPM1, TTN
**Extracellular matrix and adhesion**	COL5A1, FN1, SPARC
**Biosynthesis**	BOP1, EEF1A1, ENPP7, FKBP7, RPL23, RPL39, RPLP1, RPLP2, RPS16, RPS25, SERPINE1
**Signal transduction**	C5orf13, IQCG, IQSEC1, TSNAX
**Development**	DACT1, PDLIM7

### AdMSCs and FBs Exhibit Cellular ‘Memory’

#### AdMSCs and FBs become more similar upon induction of differentiation

Gene expression patterns of AdMSCs and FBs become more similar upon differentiation, but they still remain distinguishable within differentiated cell clusters indicating that cells ‘remember’ their origin (Figure 3A and B). We asked the question how different are gene expression patterns of AdMSC- and FB-derived cell lineages, and whether the differences vary according to cell lineages. Undifferentiated cells together with lineage-specific samples were included in the ANOVA to find differentially expressed genes (FDR of 1%) between AdMSC- and FB-derived adipocytes, osteoblasts and chondrocytes. 45 genes were found to be differentially expressed between AdMSC- and FB-derived adipocytes (Figure 4B). For AdMSC- and FB-derived osteoblasts or chondrocytes the number of differentially expressed genes was 215 and 104, respectively. This result first confirms that differences between different cell types (792 genes) are greater than differences between AdMSC- and FB-derived cells. Secondly, the fact that more genes were differentially expressed between AdMSC- and FB-derived osteoblasts and chondrocytes than between AdMSC- and FB-derived adipocytes, indicates that AdMSCs and FBs became more similar upon adipogenic differentiation. It suggests that switch of stromal cell regulatory mechanisms into adipocyte-specific regulation requires less time than switch into osteoblast- and chondrocyte-specific regulation.

#### AdMSC- and FB-derived cells exhibit distinct gene expression

To answer the question whether genes that are initially distinctly expressed in AdMSCs and FBs also remain differentially expressed in differentiated cells, the comparison of genes differentially expressed in undifferentiated and differentiated AdMSCs and FBs was done and the extent of overlap was determined for each AdMSC- and FB-derived differentiated cell type. Results of the analysis were visualized using Venn diagram, where the size of a circle is proportional to the number of genes it represents (Figure 4B). A fraction of distinctly expressed genes between undifferentiated AdMSCs and FBs (light grey) stay distinct in the differentiated cells (light blue), but also many differentiation-related genes become differently expressed in the AdMSC- and FB-derived cells (yellow, pink or blue) as shown in Figure 4B. Interestingly, the number of genes that become different in adipocytes (33 genes) is smaller than in other differentiated cells (179 genes in osteoblasts; 82 genes in chondrocytes). Also, equally small number of genes remains distinctly expressed between AdMSCs and FBs upon adipogenic induction (12 genes), which is less evident upon osteogenic (36 genes) and chondrogenic (22 genes) induction. This result confirms that AdMSC- and FB-derived adipocytes are more alike than other AdMSC- and FB-derived cells. Further, osteogenic differentiation has the smallest effect on the regulation of genes that are initially differently expressed between undifferentiated AdMSCs and FBs compared with adipogenic and chondrogenic differentiation. Taken together, the differences between AdMSC- and FB-derived differentiated cells originate from both the initially distinct gene expression patterns and gene expression acquired in the process of differentiation.

#### AdMSCs and FBs express cellular ‘memory’ genes

The fact that several genes that are differently expressed in AdMSCs and FBs remain differently expressed in AdMSC- and FB-derived differentiated cells raises the possibility that the cells express so called ’source’-specific cell ’memory’ genes that are not regulated during the differentiation. Our data show that high expression of *COL1A1*, *COL1A2*, *EFEMP1 (fibulin 3)*, *FN1 (fibronectin 1)*, *GGT5 (gamma-glutamyltransferase 5)* and *TPM2 (tropomyosin 2)* is characteristic for AdMSCs and AdMSC-derived cells. On the other hand, expression of *S100A4 (fibroblast-specific protein 1)* and *TK1 (thymidine kinase 1)* is characteristic for FBs and FB-derived cell types. It would be of interest to learn whether after longer period of differentiation the differential expression of those ’memory’ genes in AdMSC- and FB-derived mature adipocytes, osteoblasts and chondrocytes will remain present or disappear.

### AdMSCs and FBs Exhibit Similar Dynamics of Adipogenic and Osteogenic Differentiation but Distinct Dynamics of Chondrogenic Differentiation

#### Lineage-specific gene regulation occurs early in differentiation and persists over time

It is well known that cell differentiation is a process of sequential induction of regulatory genes that in turn initiate the expression of a pile of tissue-specific target genes. Still, each developmental process requires the activation of a specific transcriptional program. Our data show that global changes in cell type-specific gene expression take place quickly upon differentiation of AdMSCs and FBs (Figure 3C). Next we performed more detailed analysis of dynamics of differentiation of AdMSCs and FBs into adipocytes, osteoblasts and chondrocytes.

**Figure 5:**
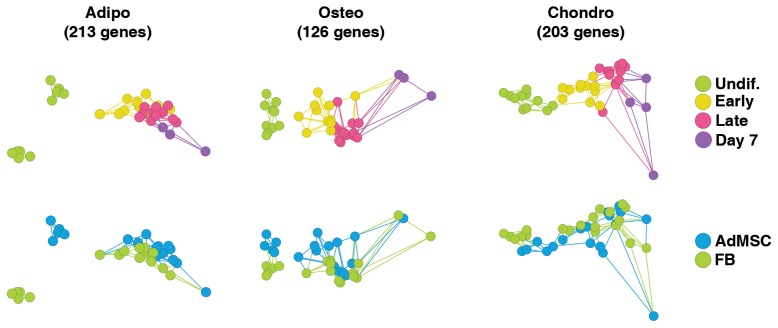
PCA of lineage-regulated gene expression. ANOVA with FDR of 1% between different time points recovered 213 genes in adipogenesis, 126 genes in osteogenesis and 203 genes in chondrogenesis that were regulated over time. These genes were used to visualize the samples in a PCA plot. Major changes in gene expression occur early in differentiation and persist over time. Abbreviations: Undif., undifferentiated; Adipo, adipocyte; Osteo, osteoblast; Chondro, chondrocyte; AdMSC, adipose-derived mesenchymal stem cell; FB, fibroblast.

To visualize transcriptome profiles of differentiating AdMSCs and FBs along adipogenic, osteogenic and chondrogenic lineages over time, the daily time points except day 0 as ‘undif.’ and ‘day 7’, were assembled into the following groups: ‘early’ including days 1–3 and ‘late’ including days 4–6. Gene expression at different time points was compared using ANOVA (FDR of 1%) and significantly differentially expressed genes were used to visualize the linkage of different samples in the PCA plot based on time group, and AdMSC or FB annotations (Figure 5). In total, 213 lineage-specific genes were found to be regulated over time during adipogenesis, 126 during osteogenesis and 203 during chondrogenesis. The genes are listed in Table S2. AdMSCs and FBs differentiated into adipocytes, osteoblasts and chondrocytes cluster together or are connected with each other through edges with little effect of time. In contrast, the genes that were regulated over time clearly placed undifferentiated cells into separate clusters that have no edge-connections with differentiated samples, except for undifferentiated AdMSCs that were related to ‘early’ chondrocytes. Hence, it reveals that major changes in lineage-specific gene expression occur early in differentiation and persist over time.

#### Gene expression dynamics upon chondrogenic differentiation is different between AdMSCs and FBs

**Figure 6:**
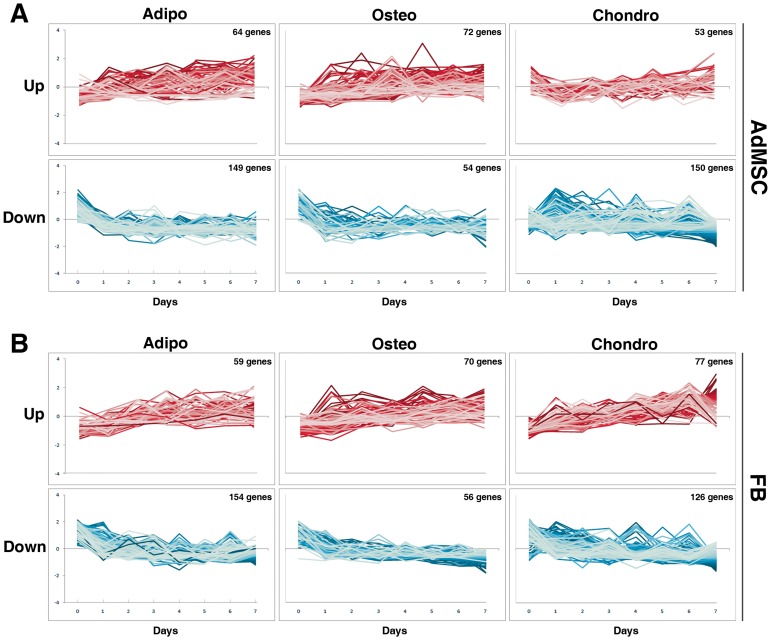
Gene expression dynamics. The expression dynamics of lineage-regulated genes in A) AdMSCs and B) FBs was visualized using line plots. The scale on y-axis shows the up or down regulation in standard deviations from the mean expression for each gene. AdMSCs and FBs share similar gene expression dynamics - quick down regulation (lower panels, blue) but slow up regulation (upper panels, red) in gene expression along adipogenesis and osteogenesis. However, the dynamics of chondrogenesis differs between AdMSCs and FBs. Abbreviations: Adipo, adipocyte; Osteo, osteoblast; Chondro, chondrocyte; AdMSC, adipose-derived mesenchymal stem cell; FB, fibroblast.

The analysis of above described gene expression data shows that approximately 70% of adipogenesis-related and 43% of osteogenesis-related genes are down regulated in the process of differentiation of both AdMSCs and FBs (Table S3). These results show that gene repression is the major mechanism of differentiation of adipocytes, whereas osteogenic differentiation is accompanied by smaller changes in global gene expression with slightly more genes up regulated (57%) than down regulated during differentiation. Chondrogenesis-related genes show different expression patterns in AdMSCs and FBs (Table S3). More genes were down regulated in AdMSCs (74%) upon chondrogenic differentiation than in FBs (62%). Next we analyzed whether the up and down regulation of gene expression occured similarly over time. The scale in line plots (Figure 6) shows gene regulation in standard deviations from the mean expression for each gene. Down regulation in gene expression was quick but up regulation occurred slowly over the week upon adipogenic and osteogenic differentiation of AdMSCs and FBs (Figure 6A and B). Interestingly, AdMSCs and FBs exhibited distinct gene expression dynamics upon chondrogenesis. Smaller but bidirectional changes in gene regulation occurred in AdMSCs throughout chondrogenesis, whereas in FBs a transient down-regulation in gene expression was followed by constant up-regulation along chondrogenic differentiation. This observation confirms that the transcriptome profiles of AdMSCs and chondrocytes are more alike and less changes in gene expression need to occur in AdMSCs than in FBs to become chondrocytes.

## Discussion

Stem cells are promising tools to study mechanisms of development and regeneration. Molecular characterization of MSCs is held back by the lack of marker genes that would distinguish them from other cell types in different tissues. MSCs are similar to FBs in growth properties, morphology, surface marker expression and developmental potential as well as origin. The global gene expression analysis of AdMSCs and FBs, both in the undifferentiated state and in the process of differentiation along adipogenic, osteogenic and chondrogenic lineages using cells from the same donors, allowed the identification of cell type-specific gene expression dynamics of two closely related stromal stem cells.

First, our study reveals that the transcriptome profiles of undifferentiated AdMSCs and FBs are distinct and stay distinct upon differentiation despite the similar general characteristics of the cells. In previous studies the comparison of gene expression profiles between AdMSCs and FBs has been carried out using cells from different donors, body locations and developmental stage (eg fetal or adult tissues), leading to possible variation in gene expression that is not directly related to the differences between these cell types [8], [9], [20]. Independently-derived hESC lines were identified to exhibit unique gene expression signature due to high genetic variability [21], [22]. Moreover, different MSC populations have been shown to exhibit a unique genomic signature [23]. We found, that the global gene expression patterns differ between AdMSCs and FBs derived from matching donors. Differences between AdMSCs and FBs did not disappear completely upon one week of differentiation probably due to the slow proccess of transition of the original cell to another cell type. In fact, we noticed many new differentially expressed genes to be present in AdMSC- and FB-derived differentiated cells compared with undifferentiated cells. Little attention has been paid to the comparison of gene expression profiles of differentiated cells that are derived from different progenitors but under similar differentiation conditions. Our data also indicate, that cells retain the expression of some ‘memory’ genes that trace back to the tissue origin of the cells. Similar phenomenon of cellular memory has been described for induced pluripotent stem cells (iPS). The gene expression analysis of iPS cells generated from different mature tissue types has revealed that iPS cells recall their original tissue type, although they all share similar morphology and expression of pluripotency genes [24]. However, it has been proposed that reprogramming of cells is a slow process and the memory of the cells’ origin will be erased over time [25]. It is possible then that the differences in gene expression profiles of AdMSC- and FB-derived adipocytes, osteoblasts and chondrocytes will disappear after longer differentiation.

Secondly, the analysis of gene expression profiles over time shows that the differences in lineage-specific gene expression occur early in differentiation of both AdMSCs and FBs. Interestingly, changes in the gene expression of AdMSCs and FBs upon induction are related initially to rapid down-regulation of gene expression, whereas up regulation occurs slowly over the week. It has been suggested that gene repression is a predominant early mechanism before final cell commitment and that lineage-specific molecular processes are transcriptionally up regulated only after commitment [26]. The results of our analysis support the idea that cells respond to induction of differentiation by rapidly resetting their original transcriptional program and gradually expressing lineage-associated genes. Although such general mechanism is shared by AdMSCs and FBs along differentiation into adipocytes and osteoblasts, the extent of gene repression is higher upon adipogenic induction. Notably, our findings suggest that the switch from stromal regulation to adipogenic regulation is faster than the switch to osteoblast and chondrocytes regulation.

Thirdly, dynamics of chondrogenic differentiation is different in AdMSCs and FBs. Unlike in FBs, in AdMSCs several genes that become up regulated along chondrogenesis are initially down-regulated and *vice versa*, many of those genes that become down regulated over the week, are initially up regulated upon differentiation. The distinct pattern of gene regulation upon chondrogenesis in AdMSCs could be related to the observation that AdMSCs are more similar to chondrocytes in the undifferentiated state than FBs. It is intruiging to speculate that AdMSCs are pre-committed to chondrocyte development and initiation of differentiation does not involve global transcriptional reprogramming. Such pre-commitment of AdMSCs seems not to affect their ability to differentiate into other cell types similarly with FBs. It has been shown that lineage-committed MSCs can transdifferentiate into other cell types in response to inducive extracellular cues [27]. Also, it has been proposed that uncommitted adult stem cells maintain their multipotency by expressing basal levels of genes characteristic to different lineages and that certain groups of genes are selectively suppressed upon stimulation prior to commitment to a given characteristic phenotype [28], [29]. It turns out then that AdMSCs and FBs use globally similar early mechanisms of differentiation into adipocytes and osteoblasts but exhibit distinct mechanisms of chondrogenic differentiation.

Together, our study shows that stromal stem cells including adipose-derived AdMSCs and dermal FBs exhibit distinct dynamics of differentiation into mesodermal cell types under similar experimental conditions. AdMSCs and FBs exploit similar early mechanisms for differentiation into adipocytes and osteoblasts but show different molecular mechanisms for chondrogenic differentiation. Further finding suggests that the switch from stromal regulation to adipocyte regulation is faster than the switch to osteoblast and chondrocyte regulation. The results of the global study provide relevant insight to the molecular mechanisms of differentiation of stromal stem cells that can be used in further studies.

## Materials and Methods

### Ethics Statement

Experiments with human tissues were approved by National Institute for Health Development and Ethics Committee in Estonia (Approval No 2234 from Dec 09, 2010).

### Cell Isolation and Cultivation

AdMSCs were isolated from human subcutaneous adipose tissue according to Lin et al. and Yamamoto et al. [30], [31] with slight modifications. Briefly, adipose tissue was digested with 0.1% collagenase (Gibco) in serum-free alphaMEM (a modification of Minimum Essential Medium (MEM), contains sodium pyruvate, lipoic acid, vitamin B_12_, biotin, and ascorbic acid, Gibco 32571) at 37°C for 1.5 h, followed by neutralization of enzyme activity with 20% fetal bovine serum (FBS) and 1% penicillin-streptomycin alphaMEM growth medium. Following centrifugation, stromal cell pellet was passed through a 100 µm nylon mesh (BD Biosciences) and resuspended in 10% FBS growth medium, plated at a density of 10 000 cells/cm^2^ and incubated at 37°C with 5% CO_2_. After 48 h medium was replaced to remove non-adherent cells. Further cultivation was performed under standard cell culture conditions. Fibroblasts were isolated from dermal skin of the same donors as AdMSCs, using a method described before [32]. Briefly, primary culture was established by fibroblast outgrowth from skin explants placed onto Primaria dish (BD Falcon) in 10% FBS and 1% penicillin-streptomycin DMEM-High Glucose (a modification of Eagle’s Minimal Essential Medium, contains sodium pyruvate, higher glucose levels, Gibco 10569) growth medium.

### *In vitro* Differentiation

Passage three or four cells were plated at density of 15 000 cells/cm^2^ 72 hours prior to induction of differentiation. 10% FBS and 1% penicillin-streptomycin containing growth medium was supplemented with:

- 1 µM dexamethasone, 500 µM IBMX (3-isobutyl-1methylxanthine), 100 µM indomethacin and 10 µg/ml insulin for adipogenic induction,
- 100 nM dexamethasone, 50 µM L-ascorbic acid 2-phosphate and 10 mM glycerol 2-phosphate for osteogenic induction,
- 50 µM L-ascorbic acid 2-phosphate, 6,25 µg/ml insulin and 10 ng/ml TGFbeta-1 (Peprotech) for chondrogenic induction.

Treatment media was changed once (on day 3) during the 7-day differentiation assay or twice a week during a long-term differentiation assay. All chemicals, if not specified differently, were purchased from Sigma. Accumulation of lipid droplets in adipocytes was determined by Oil Red O (ORO) staining as previously described [14]. For quantitative analysis, optical density of eluted ORO was measured at 510 nm. Osteoblasts were analyzed for the formation of calcified matrix by Alizarin Red S (ARS) staining as described in [14]. For quantitative analysis, ARS-stained cell monolayers were scraped off the dish in 10% acetic acid and optical density of the supernatant was measured at 405 nm. Chondrocyte differentiation was determined by Alcian Blue (AB) staining of proteoglycan-rich matrix. Briefly, 4% PFA-fixed cells were washed with water, incubated for 30 min at RT with 10 mg/ml AB solution in 5% acetic acid, washed 4 times with water, and photographed. For quantitative analysis, AB-stained cell monolayers were scraped off the dish in 6 M guanidine HCl and optical density of the supernatant was measured at 600 nm.

### RNA Isolation

Cells were lyzed at day 0, 1, 2, 3, 4, 5, 6 and 7 of adipogenic, osteogenic and chondrogenic differentiation for total RNA extraction using Trizol reagent (Invitrogen). Following a phenol/chloroform extraction and isopropanol precipitation, RNA samples were treated with DNase I using DNA-free ™ kit (Ambion). The resulting 96 RNA samples were applied to sample preparation for deep sequencing.

### Multiplex RNA-seq and Data Analysis

Gene expression analysis was performed as previously described for single-cells [16]. Multiplex mRNA-seq was performed using the same approach, but starting with 10 ng of total RNA instead of single cells, and using only 10 cycles of PCR for the cDNA amplification. Statistical analysis (ANOVA), hierarchical clustering and PCA were performed using the Qlucore Omics Explorer (Qlucore AB, Lund, Sweden). Selection of genes for ANOVA (Analysis of variation) was based on the false-discovery rate (FDR = q) to control for multiple testing. FDR was used as a measure of significance of the observed effects. PCA was used on ANOVA-filtered data (except Figure 2) to visualize differences between groups other than those used in the ANOVA, or within the groups used in the ANOVA (See Results section for specifications). Raw sequencing data is publically available at NCBI (GEO accession number GSE37521).

## Supporting Information

Figure S1 **Differences in gene expression of AdMSCs and FBs.** ANOVA (with FDR of 5%) between undifferentiated AdMSCs and FBs resulted in 178 differentially expressed genes, 59 with higher and 119 with lower expression in AdMSCs than in FBs. The scale shows the up (light red) or down regulation (light blue) in standard deviations from the mean expression for each gene.
(TIF)

Table S1 **The list of samples used in the study.**
(DOCX)

Table S2 **The list of lineage-specific genes.**
(XLSX)

Table S3 **Up and down regulation of genes during differentiation.**
(DOCX)
