# Supplementary figures and images for: RNA-Seq Analysis Reveals Different Dynamics of Differentiation of Human Dermis- and Adipose-Derived Stromal Stem Cells
Source: PLoS One. 2012 Jun 19;7(6):e38833. doi: 10.1371/journal.pone.0038833 (PMC3378616; doi:10.1371/journal.pone.0038833)

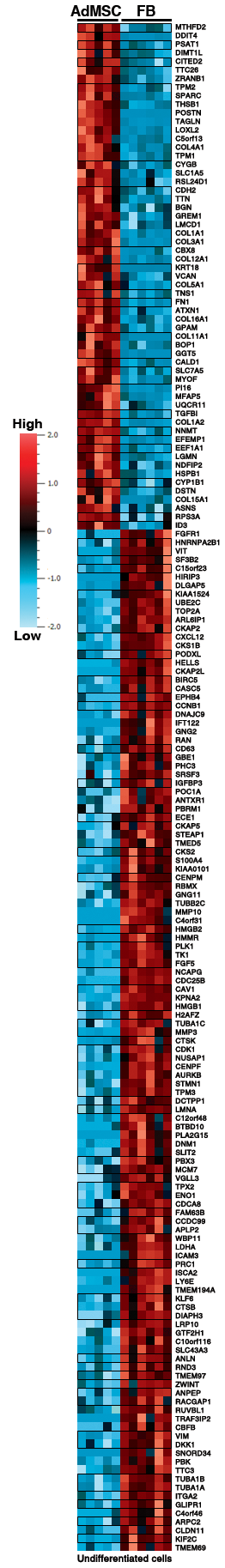

Supplement: Figure S1 — Differences in gene expression of AdMSCs and FBs. ANOVA (with FDR of 5%) between undifferentiated AdMSCs and FBs resulted in 178 differentially expressed genes, 59 with higher and 119 with lower expression in AdMSCs than in FBs. The scale shows the up (light red) or down regulation (light blue) in standard deviations from the mean expression for each gene. (TIF) [file pone.0038833.s001.tif]
