# Supplementary material for: RNA-Seq Analysis Reveals Different Dynamics of Differentiation of Human Dermis- and Adipose-Derived Stromal Stem Cells
Source: PLoS One. 2012 Jun 19;7(6):e38833. doi: 10.1371/journal.pone.0038833 (PMC3378616; doi:10.1371/journal.pone.0038833)
Supplement: Table S1 — The list of samples used in the study. (DOCX) [file pone.0038833.s002.docx]

| **Sample No** | **Cell type** | **Donor** | **Lineage** | **Time point - day** | **Sample No** | **Cell type** | **Donor** | **Lineage** | **Time point - day** |
| --- | --- | --- | --- | --- | --- | --- | --- | --- | --- |
| **1** | AdMSC | 1 | Undifferentiated | 0 | **49** | FB | 1 | Undifferentiated | 0 |
| **2** | AdMSC | 1 | Adipogenesis | 1 | **50** | FB | 1 | Adipogenesis | 1 |
| **3** | AdMSC | 1 | Adipogenesis | 2 | **51** | FB | 1 | Adipogenesis | 2 |
| **4** | AdMSC | 1 | Adipogenesis | 3 | **52** | FB | 1 | Adipogenesis | 3 |
| **5** | AdMSC | 1 | Adipogenesis | 4 | **53** | FB | 1 | Adipogenesis | 4 |
| **6** | AdMSC | 1 | Adipogenesis | 5 | **54** | FB | 1 | Adipogenesis | 5 |
| **7** | AdMSC | 1 | Adipogenesis | 6 | **55** | FB | 1 | Adipogenesis | 6 |
| **8** | AdMSC | 1 | Adipogenesis | 7 | **56** | FB | 1 | Adipogenesis | 7 |
| **9** | AdMSC | 2 | Undifferentiated | 0 | **57** | FB | 2 | Undifferentiated | 0 |
| **10** | AdMSC | 2 | Adipogenesis | 1 | **58** | FB | 2 | Adipogenesis | 1 |
| **11** | AdMSC | 2 | Adipogenesis | 2 | **59** | FB | 2 | Adipogenesis | 2 |
| **12** | AdMSC | 2 | Adipogenesis | 3 | **60** | FB | 2 | Adipogenesis | 3 |
| **13** | AdMSC | 2 | Adipogenesis | 4 | **61** | FB | 2 | Adipogenesis | 4 |
| **14** | AdMSC | 2 | Adipogenesis | 5 | **62** | FB | 2 | Adipogenesis | 5 |
| **15** | AdMSC | 2 | Adipogenesis | 6 | **63** | FB | 2 | Adipogenesis | 6 |
| **16** | AdMSC | 2 | Adipogenesis | 7 | **64** | FB | 2 | Adipogenesis | 7 |
| **17*** | AdMSC | 1 | Undifferentiated | 0 | **65** | FB | 1 | Undifferentiated | 0 |
| **18*** | AdMSC | 1 | Osteogenesis | 1 | **66** | FB | 1 | Osteogenesis | 1 |
| **19** | AdMSC | 1 | Osteogenesis | 2 | **67** | FB | 1 | Osteogenesis | 2 |
| **20** | AdMSC | 1 | Osteogenesis | 3 | **68** | FB | 1 | Osteogenesis | 3 |
| **21** | AdMSC | 1 | Osteogenesis | 4 | **69** | FB | 1 | Osteogenesis | 4 |
| **22** | AdMSC | 1 | Osteogenesis | 5 | **70** | FB | 1 | Osteogenesis | 5 |
| **23** | AdMSC | 1 | Osteogenesis | 6 | **71** | FB | 1 | Osteogenesis | 6 |
| **24*** | AdMSC | 1 | Osteogenesis | 7 | **72** | FB | 1 | Osteogenesis | 7 |
| **25** | AdMSC | 2 | Undifferentiated | 0 | **73** | FB | 2 | Undifferentiated | 0 |
| **26** | AdMSC | 2 | Osteogenesis | 1 | **74** | FB | 2 | Osteogenesis | 1 |
| **27** | AdMSC | 2 | Osteogenesis | 2 | **75** | FB | 2 | Osteogenesis | 2 |
| **28*** | AdMSC | 2 | Osteogenesis | 3 | **76** | FB | 2 | Osteogenesis | 3 |
| **29** | AdMSC | 2 | Osteogenesis | 4 | **77** | FB | 2 | Osteogenesis | 4 |
| **30** | AdMSC | 2 | Osteogenesis | 5 | **78** | FB | 2 | Osteogenesis | 5 |
| **31** | AdMSC | 2 | Osteogenesis | 6 | **79** | FB | 2 | Osteogenesis | 6 |
| **32** | AdMSC | 2 | Osteogenesis | 7 | **80** | FB | 2 | Osteogenesis | 7 |

**Table S1.** The list of samples used in the study.

| **Sample No** | **Cell type** | **Donor** | **Lineage** | **Time point - day** | **Sample No** | **Cell type** | **Donor** | **Lineage** | **Time point - day** |
| --- | --- | --- | --- | --- | --- | --- | --- | --- | --- |
| **33** | AdMSC | 1 | Undifferentiated | 0 | **81** | FB | 1 | Undifferentiated | 0 |
| **34** | AdMSC | 1 | Chondrogenesis | 1 | **82** | FB | 1 | Chondrogenesis | 1 |
| **35** | AdMSC | 1 | Chondrogenesis | 2 | **83** | FB | 1 | Chondrogenesis | 2 |
| **36** | AdMSC | 1 | Chondrogenesis | 3 | **84** | FB | 1 | Chondrogenesis | 3 |
| **37** | AdMSC | 1 | Chondrogenesis | 4 | **85** | FB | 1 | Chondrogenesis | 4 |
| **38** | AdMSC | 1 | Chondrogenesis | 5 | **86** | FB | 1 | Chondrogenesis | 5 |
| **39** | AdMSC | 1 | Chondrogenesis | 6 | **87** | FB | 1 | Chondrogenesis | 6 |
| **40*** | AdMSC | 1 | Chondrogenesis | 7 | **88** | FB | 1 | Chondrogenesis | 7 |
| **41** | AdMSC | 2 | Undifferentiated | 0 | **89** | FB | 2 | Undifferentiated | 0 |
| **42** | AdMSC | 2 | Chondrogenesis | 1 | **90** | FB | 2 | Chondrogenesis | 1 |
| **43** | AdMSC | 2 | Chondrogenesis | 2 | **91** | FB | 2 | Chondrogenesis | 2 |
| **44** | AdMSC | 2 | Chondrogenesis | 3 | **92** | FB | 2 | Chondrogenesis | 3 |
| **45** | AdMSC | 2 | Chondrogenesis | 4 | **93** | FB | 2 | Chondrogenesis | 4 |
| **46** | AdMSC | 2 | Chondrogenesis | 5 | **94** | FB | 2 | Chondrogenesis | 5 |
| **47** | AdMSC | 2 | Chondrogenesis | 6 | **95** | FB | 2 | Chondrogenesis | 6 |
| **48** | AdMSC | 2 | Chondrogenesis | 7 | **96** | FB | 2 | Chondrogenesis | 7 |

*Samples that were removed from gene expression analysis
