# Supplementary material for: RNA-Seq Analysis Reveals Different Dynamics of Differentiation of Human Dermis- and Adipose-Derived Stromal Stem Cells
Source: PLoS One. 2012 Jun 19;7(6):e38833. doi: 10.1371/journal.pone.0038833 (PMC3378616; doi:10.1371/journal.pone.0038833)
Supplement: Table S3 — Up and down regulation of genes during differentiation. (DOCX) [file pone.0038833.s004.docx]

**Table S3.** Up and down regulation of genes during differentiation.

| **Gene regulation** | **Adipo** | | **Osteo** | | **Chondro** | |
| --- | --- | --- | --- | --- | --- | --- |
|  | AdMSC | FB | AdMSC | FB | AdMSC | FB |
| Up | 30% | 28% | 57% | 56% | 26% | 38% |
| Down | 70% | 72% | 43% | 44% | 74% | 62% |
| **Total** | **213** | | **126** | | **203** | |
